# CNS relapse in patients with DLBCL treated with lenalidomide plus R-CHOP (R2CHOP): analysis from two phase 2 studies

**DOI:** 10.1038/s41408-018-0097-0

**Published:** 2018-06-26

**Authors:** Ayed O. Ayed, Annalisa Chiappella, Levi Pederson, Betsy R. Laplant, Angela Giovanna Congiu, Gianluca Gaidano, Michele Spina, Alessandro Re, Federica Cavallo, Gerardo Musuraca, William R. Macon, Thomas Witzig, Umberto Vitolo, Grzegorz S. Nowakowski

**Affiliations:** 1 Division of Hematology, Mayo Clinic, Rochester, MN USA; 2 Division of Hematology, Città della Salute e della Scienza Hospital and University, Torino, Italy; 3 Department of Health Sciences Research, Mayo Clinic, Rochester, MN USA; 4 Department of Hematology, IRCCS San Martino Hospital and University, Genova, Italy; 5 Division of Hematology, Department of Translational Medicine, Amedeo Avogadro University of Eastern Piedmont, Novara, Italy; 6 Division of Medical Oncology A, National Cancer Institute, Aviano, Italy; 7 Department of Hematology, ASST Spedali Civili di Brescia, Brescia, Italy; 8 Department of Hematology, University of Torino, Torino, Italy; 9 Department of Hematology, Istituto Scientifico Romagnolo Per Lo Studio E La Cura Dei Tumori, Meldola (FC), Meldola, Italy; 10 Department of Laboratory Medicine and Pathology, Mayo Clinic, Rochester, MN USA

## Abstract

Central nervous system (CNS) relapse of diffuse large B-cell lymphoma (DLBCL) is a devastating event occurring in ~ 5% of patients treated with R-CHOP. We hypothesized that adding lenalidomide to R-CHOP (R2CHOP) may decrease the risk of CNS relapse. We analyzed records for patients with DLBCL from two R2CHOP trials. We assessed variables pertinent to the CNS-International Prognostic Index (CNS-IPI) scoring system and classified patients into groups of low, intermediate, and high risk of CNS relapse. The 2-year CNS relapse rate for each risk group was estimated using the Kaplan–Meier method and compared with reported rates in cohorts treated with contemporary chemoimmunotherapy. A total of 136 patients were included. Mean age was 65 and median follow-up was 48.2 months. 10.3, 71.3, and 18.4% of patients were classified into low, intermediate, and high-risk CNS-IPI groups, respectively. Only one of 136 patients developed CNS relapse, corresponding to an incidence of 0.7% and an estimated 2-year CNS relapse rate of 0.9% for the entire R2CHOP cohort. The estimated 2-year CNS relapse rates for the low, intermediate, and high-risk groups were 0, 0, and 5.0%, respectively. Frontline therapy with R2CHOP in patients with DLBCL is associated with a lower-than-expected rate of CNS relapse.

## Introduction

Relapse of aggressive non-Hodgkin lymphoma (NHL) in the central nervous system (CNS) is an uncommon but serious and potentially fatal event. Incidence rates of up to 10% have been reported^1–4^. The addition of rituximab to standard CHOP therapy (R-CHOP; cyclophosphamide, doxorubicin, vincristine, prednisone) has contributed to a reduction in both systemic and CNS relapse^5–7^. It is important to note however that the blood–brain barrier (BBB) renders the CNS a sanctuary site where penetration by standard chemoimmunotherapy is insufficient. Given the poor outcome of CNS relapse, there is clearly a need to incorporate agents that cross the BBB in upfront regimens to mitigate the risk of this event.

A number of factors are associated with an increased risk of developing CNS relapse in NHL such as older age, advanced stage, extranodal disease, elevated lactate dehydrogenase (LDH), and renal/adrenal involvement^3,8^. The CNS-International Prognostic Index (CNS-IPI) is a validated scoring system^3^ that risk-stratifies patients with diffuse large B-cell lymphoma (DLBCL) and may potentially help identify those at higher risk of developing CNS relapse.

Lenalidomide, an immunomodulatory agent that penetrates the CNS, has shown promise in relapsed/refractory (R/R) aggressive NHL, with well-demonstrated single-agent activity and tolerability^9,10^. Specifically, lenalidomide is active and well-tolerated in heavily pre-treated CNS lymphoma^11^. Combination therapy with lenalidomide (with bendamustine/rituximab (BR) or R-CHOP) has also been shown to be effective and tolerable in both the upfront and R/R settings^12–14^. Given its ability to cross the BBB, we hypothesized that lenalidomide would lower the risk of CNS relapse when included in induction therapy. In this study, we evaluated and characterized the incidence of isolated CNS relapse in a combined cohort of DLBCL patients treated with upfront lenalidomide plus R-CHOP (R2CHOP).

## Methods

Patients with histologically confirmed DLBCL treated with R2CHOP who were enrolled in two phase 2 trials—Mayo Clinic (MC)^15^ (NCT00670358) and Fondazione Italiana Linfomi (FIL)^12^ (NCT00907348)—were included in this study. Several patients (*n* = 23) in the MC cohort whose histology excluded them from the final trial analysis (owing to histologic transformation or composite lymphoma) were included in this analysis, as they were allowed to remain on study following a protocol amendment. All patients had positron emission tomography (PET) imaging prior to and after completion of therapy, but not brain magnetic resonance imaging. Accordingly, patients were deemed to have no CNS involvement at baseline by clinical assessment and PET imaging only.

Data collected from patient records included age, gender, disease stage, status at last follow-up, cell of origin (COO) based on immunohistochemistry according to Hans’ algorithm, LDH level, extranodal site involvement, Eastern Cooperative Oncology Group (ECOG) performance status, and administration of CNS prophylaxis. The protocol for each trial allowed intrathecal (IT) chemotherapy for prophylactic purposes per local practice (MC uses methotrexate 12 mg IT for 6 doses, FIL uses methotrexate 12 mg, or 15 mg IT for 4–6 doses). No systemic CNS prophylaxis was allowed.

### Risk stratification

The combined cohort of patients was stratified into three risk groups based on calculated CNS-IPI score. This scoring tool uses independent variables (age, stage, LDH level, ECOG performance status, extranodal sites, adrenal/kidney involvement) to classify patients into low (score 0–1), intermediate (score 2–3), and high (score 4–6) risk of CNS relapse^3^.

### Statistical methods

Kruskal–Wallis or chi-square test was used to compare the three risk groups in terms of various clinical variables including age, stage, COO, LDH level, extranodal site involvement, ECOG performance status, and administration of CNS prophylaxis. The Kaplan–Meier method was used for time-to-event analyses. SAS^®^ software (version 9.4 M3) was used for all statistical analyses.

### Comparison with other cohorts

We compared the Kaplan–Meier estimated 2-year CNS relapse rates of R2CHOP patients for each of the CNS-IPI risk groups (low, intermediate, and high), and also for the entire cohort, to rates published for patients treated with contemporary R-CHOP. The comparison cohorts included the R-CHOP-treated cohort of the Molecular Epidemiology Resource (MER) of the University of Iowa/Mayo Clinic Specialized Program of Research Excellence database, as reported by Thanarajsingam and colleagues^16^, as well as two large DLBCL cohorts treated with contemporary chemoimmunotherapy, (German High-Grade Non-Hodgkin Lymphoma Study Group/MabThera International Trial (DSHNHL/MInT) and British Columbia Cancer Agency (BCCA) DLBCL cohorts), as reported by Schmitz et al.^3^ Data for the R-CHOP-treated cohort of the MER was obtained through direct correspondence and discussion with the authors of that study, whereas data pertaining to the CNS relapse rates for the DSHNHL/MInT and BCCA DLBCL cohorts was obtained directly from the publication for that report.

## Results

A total of 136 patients with DLBCL (87 MC patients, 49 FIL patients) were included in this study (Table 1). Mean age was 65 and median follow-up in 104 patients that were still alive at the time of the analysis was 48.2 months (range: 2.1–88.5).

**Table 1:** Baseline patient characteristics

	FIL	MC	Total
	(*n* = 49)	(*n* = 87)	(*n* = 136)
Age			
Median	69	65	68
Range	(61.0–79.0)	(19.0–87.0)	(19.0–87.0)
Clinical stage			
2	6 (12.2%)	13 (14.9%)	19 (14.0%)
3	8 (16.3%)	21 (24.1%)	29 (21.3%)
4	35 (71.4%)	53 (60.9%)	88 (64.7%)
Cell of origin			
GCB	16 (32.7%)	43 (49.4%)	59 (43.4%)
Non-GCB	16 (32.7%)	34 (39.1%)	50 (36.8%)
NA	17 (34.7%)	10 (11.5%)	27 (19.9%)
LDH above ULN			
No	26 (53.1%)	34 (39.1%)	60 (44.1%)
Yes	23 (46.9%)	53 (60.9%)	76 (55.9%)
Extranodal sites			
0 or 1	31 (63.3%)	64 (73.6%)	95 (69.9%)
>1 site	18 (36.7%)	23 (26.4%)	41 (30.1%)
Performance status			
0	17 (34.7%)	43 (49.4%)	60 (44.1%)
1	25 (51.0%)	35 (40.2%)	60 (44.1%)
2	7 (14.3%)	9 (10.3%)	16 (11.8%)
CNS prophylaxis			
No	30 (61.2%)	86 (98.9%)	116 (85.3%)
Yes	19 (38.8%)	1 (1.1%)	20 (14.7%)
CNS-IPI score			
Low	0 (0.0%)	14 (16.1%)	14 (10.3%)
Intermediate	37 (75.5%)	60 (69.0%)	97 (71.3%)
High	12 (24.5%)	13 (14.9%)	25 (18.4%)

*GCB* germinal center B-cell, *NA* not available, *ULN* upper limit of normal, *MC* Mayo Clinic, *FIL* Fondazione Italiana Linfomi

**Fig. 1:**
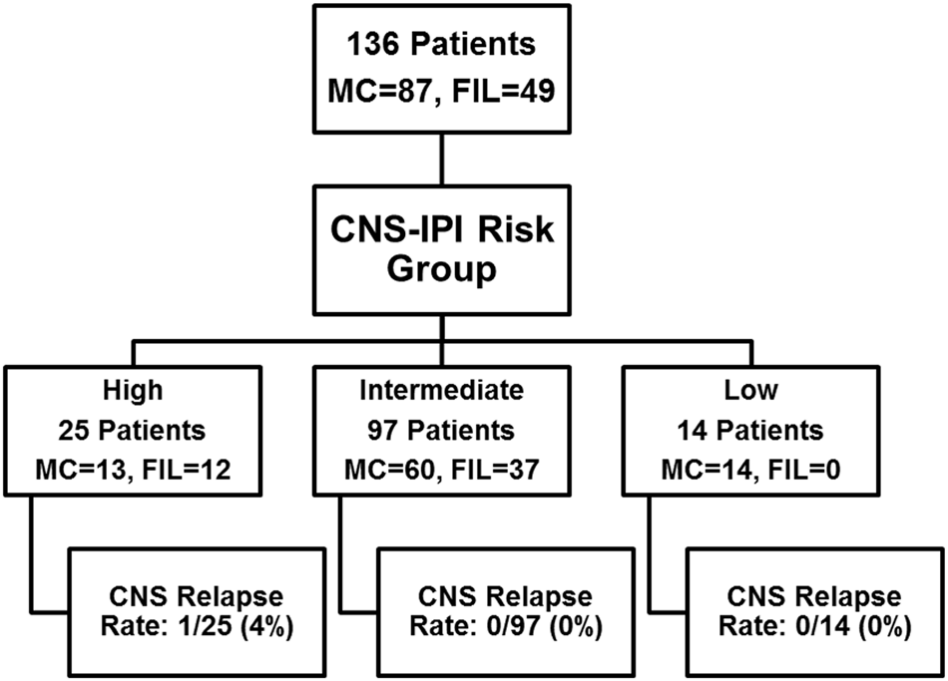
Patient flow diagram. MC = Mayo Clinic. FIL = Fondazione Italiana Linfomi

When categorized into risk groups per the CNS-IPI score, 14 (10.3%), 97 (71.3%), and 25 (18.4%) patients were classified into low, intermediate, and high-risk groups, respectively (Fig. 1). Comparison between these groups across different variables is shown in Table 2.

**Table 2:** Baseline patient characteristics by CNS-IPI score

	High risk	Intermediate risk	Low risk	*P* value
	(*n* = 25)	(*n* = 97)	(*n* = 14)	
Age				0.0014
Median	69	69	56.5	
Range	(55.0–83.0)	(19.0–87.0)	(24.0–79.0)	
Clinical stage				< 0.0001
2	0 (0.0%)	13 (13.4%)	6 (42.9%)	
3	0 (0.0%)	25 (25.8%)	4 (28.6%)	
4	25 (100.0%)	59 (60.8%)	4 (28.6%)	
Cell of origin				0.2353
GCB	7 (28.0%)	47 (48.5%)	5 (35.7%)	
Non-GCB	10 (40.0%)	35 (36.1%)	5 (35.7%)	
NA	8 (32.0%)	15 (15.5%)	4 (28.6%)	
LDH above ULN				<0.0001
No	2 (8.0%)	45 (46.4%)	13 (92.9%)	
Yes	23 (92.0%)	52 (53.6%)	1 (7.1%)	
Extranodal sites				<0.0001
0 or 1	2 (8.0%)	79 (81.4%)	14 (100.0%)	
>1 site	23 (92.0%)	18 (18.6%)	0 (0.0%)	
Performance status				<0.0001
0	5 (20.0%)	45 (46.4%)	10 (71.4%)	
1	9 (36.0%)	47 (48.5%)	4 (28.6%)	
2	11 (44.0%)	5 (5.2%)	0 (0.0%)	
CNS prophylaxis				<0.0001
No	14 (56.0%)	88 (90.7%)	14 (100.0%)	
Yes	11 (44.0%)	9 (9.3%)	0 (0.0%)	

Based on CNS-IPI score, patients are classified into low (0–1), intermediate (2–3), or high (4–6) risk of CNS relapse

*GCB* germinal center B-cell, *NA* not available, *ULN* upper limit of normal

Of the 136 patients, only one developed isolated CNS relapse (which was parenchymal in nature), yielding an incidence rate of 0.007 (0.7%). His disease at diagnosis was consistent with a GCB phenotype, extensive extranodal disease primarily involving multiple bony sites, and a calculated CNS-IPI score of 4 (high risk). Time to development of CNS relapse from diagnosis in this particular patient was 10 months and the relapse occurred 6 months after achieving a complete response (CR) by PET imaging. He was taken off protocol and salvaged with a high-dose methotrexate regimen followed by autologous stem cell transplantation.

The estimated 2-year CNS relapse rates by Kaplan–Meier method are 0.9% for the entire R2CHOP cohort and 0, 0, and 5.0% for the low, intermediate, and high-risk groups, respectively. When compared with contemporary DLBCL patients treated with R-CHOP from the MER database, the overall 2-year CNS relapse rate in the R2CHOP cohort is lower (0.9 vs. 1.8%, Table 3). Similarly, the 2-year CNS relapse rates across risk groups, and overall, are lower for the R2CHOP cohort compared with the DLBCL cohorts from DSHNHL/MInT and BCCA (Table 3), as reported by Schmitz et al.^3^.

**Table 3:** Estimated 2-year CNS relapse rate per risk group across cohorts

Two-year CNS relapse rates, estimated by Kaplan–Meier method				
Risk group	DSHNHL/MInT DLBCL cohort^a^	BCCA DLBCL cohort^a^	MER R-CHOP cohort^b^	MC/FIL R2CHOP cohort
Low	0.8% (95% CI: 0.2–1.4%)	0.8% (95% CI: 0.0–1.6%)	1.4% (95% CI: 0.5–3.7%)	0% (95% CI: 0.0–0.0%)
Intermediate	2.9% (95% CI: 1.5–4.3%)	3.9% (95% CI: 2.3–5.5%)	2.2% (95% CI: 1.2–4.2%)	0% (95% CI: 0.0–0.0%)
High	10.0% (95% CI: 5.7–14.3%)	12.0% (95% CI: 7.9–16.1%)	1.1% (95% CI: 0.2–8.1%)	5.0% (95% CI: 0.0–14.1%)
Overall	Not available	4.8% (95% CI: 3.6–6.0%)	1.8% (95% CI: 1.1–3.0%)	0.9 % (95% CI: 0.0–2.6%)

*MC/FIL* Mayo Clinic/Fondazione Italiana Linfomi, DSHNHL/MInT German High-Grade Non-Hodgkin Lymphoma Study Group/ MabThera International Trial, *BCCA* British Columbia Cancer Agency, *MER* Molecular Epidemiology Resource of the University of Iowa/Mayo Clinic Specialized Program of Research Excellence.

^a^As reported by Schmitz et al.^3^

^b^As reported by Thanarajasingam et al.^16^

## Discussion

CNS relapse in DLBCL continues to occur in a significant minority of patients and carries a poor prognosis even in the era of modern chemoimmunotherapy. Improvements in upfront regimens are needed to prevent lymphoma relapse in this sanctuary site. We report a critical observation in this analysis of a combined cohort of DLBCL patients treated with R2CHOP. Although CNS relapse rates with contemporary therapy have been roughly reported to range up to 5%^17^, we note a considerably lower rate of 0.7%. The median follow-up duration of 48.2 months is adequate to identify CNS events, as most cases of CNS relapse tend to occur within a few months from diagnosis^4^. It is worth noting that this lower-than-expected CNS relapse rate is observed in a population that is mostly intermediate and high risk (*n* = 122, 89.7%) by CNS-IPI score.

Our analysis reveals considerable practice variation with regard to the use of prophylactic IT methotrexate, with significantly more patients having received IT chemotherapy in the FIL cohort compared with the MC cohort. This reflects regional and institutional differences in the administration of CNS prophylaxis, which is often based on the provider’s judgement. This variation in practice is one of our study’s limitations since it can contribute to confounding bias. Interestingly, however, several studies have suggested that IT chemoprophylaxis is ineffective in reducing the risk of CNS relapse^1,18,19^. As such, we believe that the lack of standardization in prophylactic IT methotrexate practice did not have a significant impact on our analysis. Another limitation of our study is the relatively small number of patients in the combined cohorts. Furthermore, neither trial was powered to assess for CNS events as CNS relapse was not a particular outcome measure in the study protocols.

There continues to be significant variability and controversy with regard to optimal CNS prophylaxis strategies. This lack of consensus is driven primarily by lack of strong evidence for benefit of current strategies and the difficulty of conducting definitive randomized studies in this area^20^. Recent practice trends tend to favor systemic intravenous (IV) methotrexate over IT therapy^21,22^. Although prophylactic IV methotrexate is gaining popularity, it tends to be costly and inconvenient and has the potential for serious toxicity. Our study suggests the potential prophylactic benefit of utilizing lenalidomide, an orally bioavailable CNS-penetrating agent, when combined with R-CHOP in the upfront setting. The two phase 2 trials analyzed in this study have already demonstrated safety and manageable toxicity of such a combination.

Further assessment of the utility of lenalidomide in reducing CNS relapse risk in DLBCL is warranted. Effective stratification of patients and identification of those at higher risk will likely involve both clinical and molecular parameters. At present, two phase 3 trials evaluating the combination of lenalidomide and R-CHOP in DLBCL patients are underway (ECOG1412 (NCT01856192) and ROBUST (NCT02285062)). Characterization of CNS events in these trials will be helpful in confirming the findings we noted in this analysis.

Our finding that frontline R2CHOP may lower the risk of CNS relapse in patients with DLBCL supports an ongoing trend in clinical studies where other novel small molecules are being investigated in the upfront setting in combination with R-CHOP, such as ibrutinib (NCT01855750), venetoclax (NCT02055820), and everolimus^23^ (NCT01334502). The impact of such R(X)CHOP combinations^24^ (where X represents a novel agent molecule) on CNS relapse is yet to be defined.
